# Supplementary material for: Giant magnetocaloric effect in a rare-earth-free layered coordination polymer at liquid hydrogen temperatures
Source: Nat Commun. 2024 Oct 3;15:8559. doi: 10.1038/s41467-024-52837-x (PMC11450188; doi:10.1038/s41467-024-52837-x)
Supplement: Supplementary file 1 — Supplementary Information [file 41467_2024_52837_MOESM1_ESM.pdf]

# SUPPLEMENTARY INFORMATION FOR: Giant Magnetocaloric Effect in a Rare-Earth-Free Layered Coordination Polymer at Liquid Hydrogen Temperatures

J.J.B. Levinsky[1,2], B. Beckmann[3], T. Gottschall[4], D. Koch[5], M. Ahmadi[1], O. Gutfleisch[3], G.R. Blake[1*]

[1] Zernike Institute for Advanced Materials, University of Groningen, Nijenborgh 4, 9747AG Groningen, The Netherlands
[2] School of Chemistry and Centre for Science at Extreme Conditions, University of Edinburgh, Joseph Black building, David Brewster road, EH9 3FJ, Edinburgh, United Kingdom
[3] Functional Materials, Institute of Materials Science, Technical University of Darmstadt, Darmstadt 64287, Germany
[4] Dresden High Magnetic Field Laboratory (HLD-EMFL), Helmholtz-Zentrum Dresden-Rossendorf (HZDR), Dresden 01328, Germany
[5] Structure Research, Institute of Materials Science, Technical University of Darmstadt, Darmstadt 64287, Germany

## Section 1: Fourier-transform infrared spectroscopy

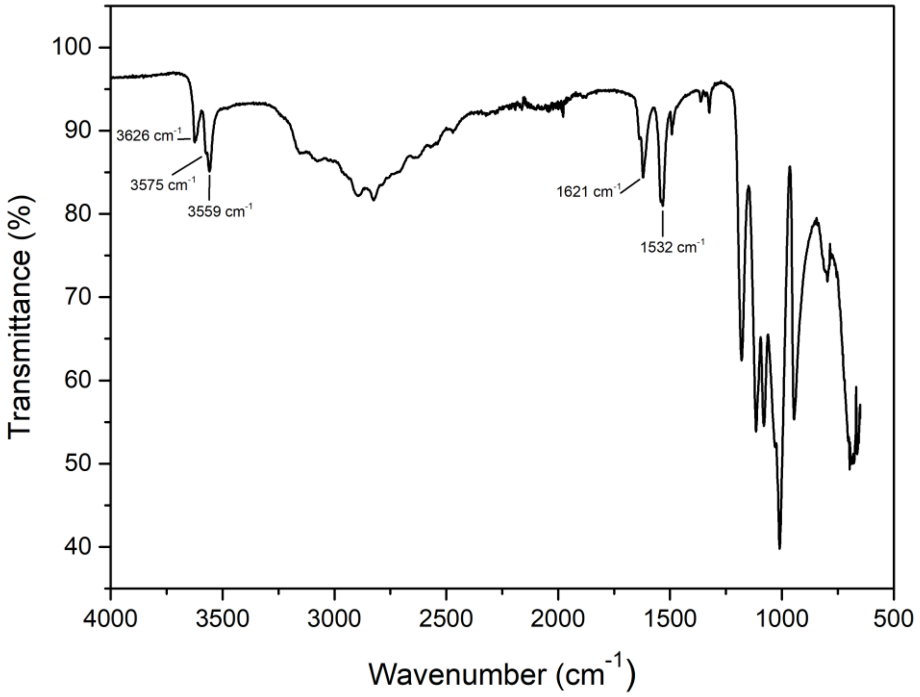


**Fig. S1 | Fourier-transform infrared spectrum for $Co_4(OH)_6(SO_4)_2[enH_2]$.**

The sharp peaks at 3626, 3575 and 3559 cm$^{-1}$ are assigned to O-H and primary amine N-H stretching vibrations. The broad set of peaks between 3250 – 2250 cm$^{-1}$ are assigned to a combination of hydrogen bonded O-H and C-H stretching vibrations. The two sharp peaks at 1621 and 1532 cm$^{-1}$ are assigned to $NH_2$ scissoring bending modes. The set of peaks observed between 1250 and 1000 cm$^{-1}$ are assigned to a combination of C-N and S=O stretching vibrations.

## Section 2: Single crystal x-ray diffraction

All non-hydrogen atoms were refined using anisotropic displacement parameters. Two twin domains were included in the model, related by 180° rotation around [110], with refined fractions of 0.725 and 0.275. The resulting refinement parameters, fractional coordinates, equivalent isotropic and anisotropic displacement parameters, bond distances and bond angles are listed in Tables S1-S5.

**Table S1 | Structure refinement parameters for $Co_4(OH)_6(SO_4)_2[enH_2]$. The numbers in parentheses indicate the estimated standard deviation.**

| | |
|---|---|
| **Temperature (K)** | 107(2) |
| **Formula** | $Co_4(OH)_6(SO_4)_2(C_2N_2H_{10})$ |
| **Formula weight (g/mol)** | 592.02 |
| **Crystal Size (mm$^3$)** | 0.2 × 0.140 × 0.04 |
| **Crystal color** | Pink |
| **Crystal habit** | Thick plate |
| **Crystal system** | Triclinic |
| **Space group** | $P\bar{1}$ (no. 2) |
| ***Z*** | 2 |
| **Density (calculated) (g/cm$^3$)** | 2.789 |
| ***F*(000)** | 588 |
| ***a* (Å)** | 8.3559(9) |
| ***b* (Å)** | 8.3653(9) |
| ***c* (Å)** | 10.5382(12) |
| ***α* (°)** | 74.944(4) |
| ***β* (°)** | 84.476(4) |
| ***γ* (°)** | 83.525(4) |
| **Volume (Å$^3$)** | 705.07 |
| **$\mu$ (mm$^{-1}$)** | 5.001 |
| **Transmission min/max** | 0.537/0.748 |
| **θ range (degrees)** | 5.064 to 66.282 |
| **Index ranges** | $-12 \le h \le 12$<br>$-11 \le k \le 12$<br>$0 \le l \le 16$ |
| **Data/restraints/parameters** | 5424 / 0 / 221 |
| **GOOF of $F^2$** | 1.044 |
| **# total reflections** | 8946 |
| **# unique reflections** | 5424 |
| **# observed $Fo > 4\sigma(Fo)$** | 4441 |
| **$R_{int}$ $I > 3\sigma(I)$** | 0.0416 |
| **$R_{sigma}$** | 0.0370 |
| **$R1$ [$Fo > 4\sigma(Fo)$]** | 0.0398 |
| **$R1$ [all data]** | 0.0577 |
| **$wR2$ [$Fo > 4\sigma(Fo)$]** | 0.1083 |
| **$wR2$ [all data]** | 0.1211 |
| **Largest peak/hole (e/Å$^3$)** | 0.77/-1.60 |

**Table S2 | Fractional atomic coordinates and equivalent isotropic displacement parameters (Å$^2$) for non-hydrogen atoms. The numbers in parentheses indicate the estimated standard deviation.**

| Atom | *x* | *y* | *z* | *U*(eq) |
|---|---|---|---|---|
| Co1 | 0.36846(5) | 0.88232(4) | -0.00767(4) | 0.00464(9) |
| Co2 | 0.5 | 0.5 | 0 | 0.00445(12) |
| Co3 | 0 | 1 | 0 | 0.00453(12) |
| Co4 | 0.12945(5) | 0.61623(4) | 0.00667(4) | 0.00456(9) |
| Co5 | 0.74863(4) | 0.74754(4) | 0.00913(4) | 0.00438(9) |
| S1 | 0.50264(8) | 0.62117(8) | 0.27894(6) | 0.00532(12) |
| S2 | 0.01996(8) | 1.1312(8) | 0.27483(6) | 0.00492(12) |
| O1 | 0.2067(3) | 1.0806(3) | -0.10146(19) | 0.0063(3) |
| O2 | 0.4065(3) | 1.0604(3) | 0.0907(2) | 0.006(4) |
| O3 | 0.184(3) | 1.1374(3) | 0.3171(2) | 0.0093(4) |
| O4 | 0.5236(3) | 0.6702(3) | 0.12962(18) | 0.0056(3) |
| O5 | 0.2921(3) | 0.4143(3) | 0.09721(19) | 0.0055(3) |
| O6 | 0.0323(3) | 1.1711(3) | 0.12562(18) | 0.0068(4) |
| O7 | 0.3283(3) | 0.6491(3) | 0.3182(2) | 0.0104(4) |
| O8 | 0.5618(3) | 0.4477(3) | 0.3275(2) | 0.0123(4) |
| O9 | 0.5906(3) | 0.7333(3) | 0.3286(2) | 0.0111(4) |
| O10 | 0.3456(3) | 0.6923(3) | -0.0955(2) | 0.0055(4) |
| O11 | 0.1541(3) | 0.8039(3) | 0.0919(2) | 0.0064(4) |
| O12 | -0.092(3) | 0.5547(3) | 0.0974(2) | 0.0062(4) |
| O13 | -0.0897(3) | 1.2624(2) | 0.3148(2) | 0.0095(4) |
| O14 | -0.0378(3) | 0.9679(3) | 0.3297(2) | 0.0103(4) |
| N1 | 0.6715(3) | 0.129(3) | 0.5014(2) | 0.0083(5) |
| C1 | 0.55(4) | 0.0556(3) | 0.4453(3) | 0.0087(5) |
| N2 | 0.1964(3) | 0.3723(3) | 0.4773(2) | 0.0096(4) |
| C2 | 0.0533(4) | 0.4387(4) | 0.5482(3) | 0.0103(5) |

**Table S3 | Anisotropic displacement parameters (Å$^2$ × 10$^3$) for non-hydrogen atoms. The numbers in parentheses indicate the estimated standard deviation.**

| Atom | $U_{11}$ | $U_{22}$ | $U_{33}$ | $U_{23}$ | $U_{13}$ | $U_{12}$ |
|---|---|---|---|---|---|---|
| Co1 | 3.6(2) | 4.0(2) | 6.51(16) | -1.65(14) | -0.04(15) | -0.73(13) |
| Co2 | 3.1(3) | 3.8(3) | 6.3(3) | -1.1(2) | -0.2(2) | -0.38(19) |
| Co3 | 3.5(3) | 3.9(3) | 6.1(3) | -1.1(2) | -0.3(2) | -0.53(19) |
| Co4 | 3.7(2) | 3.9(2) | 6.35(16) | -1.70(14) | -0.17(15) | -0.74(13) |
| Co5 | 3.7(2) | 3.9(2) | 5.74(17) | -1.68(15) | -0.51(15) | -0.23(14) |
| S1 | 6.3(3) | 5.3(3) | 4.4(2) | -1.5(2) | -0.3(2) | 0.0(2) |
| S2 | 5.7(3) | 4.2(3) | 4.8(2) | -0.9(2) | -0.2(2) | -0.8(2) |
| O1 | 4.9(10) | 7.7(10) | 5.4(8) | -0.6(7) | 0.9(7) | -0.5(7) |
| O2 | 4.1(10) | 8.5(10) | 4.8(8) | -0.4(7) | 0.3(7) | -1.2(7) |
| O3 | 6.8(10) | 12.1(10) | 9.2(9) | -1.4(7) | -3.3(7) | -1.7(8) |
| O4 | 4.0(9) | 7.4(9) | 4.6(8) | -0.7(7) | 1.0(7) | -0.4(7) |
| O5 | 4.6(10) | 6.3(9) | 5.2(8) | -1.0(7) | 0.0(7) | 0.6(7) |
| O6 | 7.7(10) | 8.4(10) | 3.8(8) | -0.9(7) | 0.4(7) | -0.7(7) |
| O7 | 6.8(9) | 15.2(10) | 7.9(9) | -1.6(8) | 2.0(7) | -0.6(8) |
| O8 | 16.7(11) | 7.4(9) | 9.5(9) | 1.3(7) | -0.3(8) | 3.5(8) |
| O9 | 13.9(11) | 13.6(10) | 7.8(9) | -3.6(8) | -2.2(8) | -5.8(8) |
| O10 | 3.9(9) | 6.9(9) | 5.0(9) | -0.9(7) | -0.2(7) | 0.5(7) |
| O11 | 6.9(10) | 8.3(10) | 3.5(9) | -0.7(8) | -0.1(8) | -1.2(8) |
| O12 | 5.3(10) | 6.5(10) | 5.6(8) | -0.3(7) | 0.9(7) | 0.1(7) |
| O13 | 11.9(10) | 8.0(9) | 7.7(9) | -2.8(7) | 2.9(7) | 2.1(8) |

| | | | | | | |
|---|---|---|---|---|---|---|
| O14 | 14.5(11) | 7.8(9) | 7.8(9) | -0.5(7) | 1.5(8) | -3.4(8) |
| N1 | 9.1(11) | 7.1(10) | 8.8(11) | -1.7(8) | -1.6(9) | -1.6(9) |
| C1 | 8.7(12) | 9.0(12) | 8.3(11) | -0.8(9) | -0.4(9) | -3.5(10) |
| N2 | 7.6(11) | 10.6(11) | 11.2(10) | -4.1(9) | -1.7(9) | 1.2(9) |
| C2 | 9.4(12) | 10.0(12) | 9.7(11) | -1.2(9) | 1.5(9) | 1.7(10) |

**Table S4 | Selected bond distances for non-hydrogen atoms**

| Bond distances $Co_4(OH)_6(SO_4)_2[enH_2]$ | | | | | | |
|---|---|---|---|---|---|---|
| **Atom** | **Atom** | **Distance (Å)** | | **Atom** | **Atom** | **Distance (Å)** |
| Co1 | O1 | 2.111(2) | | Co4 | O11 | 2.038(2) |
| Co1 | O2[1] | 2.049(2) | | Co4 | O12 | 2.055(3) |
| Co1 | O2 | 2.089(2) | | Co4 | O12[4] | 2.080(2) |
| Co1 | O4 | 2.331(2) | | Co5 | O1[1] | 2.018(2) |
| Co1 | O10 | 2.071(2) | | Co5 | O2[1] | 2.070(2) |
| Co1 | O11 | 2.070(3) | | Co5 | O4 | 2.226(2) |
| Co2 | O4[2] | 2.249(2) | | Co5 | O5[2] | 2.042(2) |
| Co2 | O4 | 2.249(2) | | Co5 | O6[1] | 2.260(2) |
| Co2 | O5[2] | 2.034(2) | | Co5 | O12[5] | 2.053(2) |
| Co2 | O5 | 2.034(2) | | S1 | O4 | 1.517(2) |
| Co2 | O10 | 2.051(2) | | S1 | O7 | 1.485(2) |
| Co2 | O10[2] | 2.051(2) | | S1 | O8 | 1.454(2) |
| Co3 | O1[3] | 2.028(2) | | S1 | O9 | 1.476(2) |
| Co3 | O1 | 2.028(2) | | S2 | O3 | 1.491(2) |
| Co3 | O6[3] | 2.239(2) | | S2 | O6 | 1.516(2) |
| Co3 | O6 | 2.239(2) | | S2 | O13 | 1.470(2) |
| Co3 | O11[3] | 2.056(2) | | S2 | O14 | 1.455(2) |
| Co3 | O11 | 2.056(2) | | N1 | C1 | 1.484(3) |
| Co4 | O5 | 2.123(2) | | C1 | C1[6] | 1.525(5) |
| Co4 | O6[3] | 2.338(2) | | N2 | C2 | 1.481(4) |
| Co4 | O10 | 2.087(3) | | C2 | C2[7] | 1.518(6) |

Symmetry related positions: [1]1-*x*,2-*y*,-*z*; [2]1-*x*,1-*y*,-*z*; [3]-*x*,2-*y*,-*z*; [4]-*x*,1-*y*,-*z*; [5]1+*x*,+*y*,+*z*; [6]1-*x*,-*y*,1-*z*; [7]-*x*,1-*y*,1-*z*

**Table S5 | Selected bond angles for non-hydrogen atoms**

| Bond angles $Co_4(OH)_6(SO_4)_2[enH_2]$ | | | | | | | | |
|---|---|---|---|---|---|---|---|---|
| **Atom** | **Atom** | **Atom** | **Angle (degrees)** | | **Atom** | **Atom** | **Atom** | **Angle (degrees)** |
| O1 | Co1 | O4 | 170.07(7) | | O1[1] | Co5 | O2[1] | 82.93(9) |
| O2[1] | Co1 | O1 | 105.67(10) | | O1[1] | Co5 | O4 | 97.03(8) |
| O2 | Co1 | O1 | 80.24(9) | | O1[1] | Co5 | O5[2] | 175.76(8) |
| O2[1] | Co1 | O2 | 80.73(9) | | O1[1] | Co5 | O6[1] | 85.37(8) |
| O2 | Co1 | O4 | 93.80(8) | | O1[1] | Co5 | O12[5] | 99.33(10) |
| O2[1] | Co1 | O4 | 80.95(8) | | O2[1] | Co5 | O4 | 83.09(8) |
| O2[1] | Co1 | O10 | 97.64(8) | | O2[1] | Co5 | O6[1] | 94.17(8) |
| O2[1] | Co1 | O11 | 173.61(9) | | O4 | Co5 | O6[1] | 176.08(7) |
| O10 | Co1 | O1 | 104.70(10) | | O5[2] | Co5 | O2[1] | 94.65(9) |
| O10 | Co1 | O2 | 175.06(8) | | O5[2] | Co5 | O4 | 86.11(8) |
| O10 | Co1 | O4 | 81.33(9) | | O5[2] | Co5 | O6[1] | 91.34(8) |
| O11 | Co1 | O1 | 80.58(10) | | O5[2] | Co5 | O12[5] | 82.94(9) |
| O11 | Co1 | O2 | 99.33(8) | | O12[5] | Co5 | O2[1] | 176.56(7) |
| O11 | Co1 | O4 | 92.68(8) | | O12[5] | Co5 | O4 | 99.15(9) |
| O11 | Co1 | O10 | 81.77(8) | | O12[5] | Co5 | O6[1] | 83.46(9) |

| | | | | | | | | |
|---|---|---|---|---|---|---|---|---|
| O4$^{2}$ | Co2 | O4 | 180.0 | | O7 | S1 | O4 | 108.00(12) |
| O5 | Co2 | O4 | 94.31(7) | | O8 | S1 | O4 | 109.52(12) |
| O5 | Co2 | O4$^{2}$ | 85.69(8) | | O8 | S1 | O7 | 110.98(13) |
| O5$^{2}$ | Co2 | O4 | 85.69(7) | | O8 | S1 | O9 | 112.17(13) |
| O5$^{2}$ | Co2 | O4$^{2}$ | 94.31(8) | | O9 | S1 | O4 | 107.91(14) |
| O5 | Co2 | O5$^{2}$ | 180.0 | | O9 | S1 | O7 | 108.12(13) |
| O5$^{2}$ | Co2 | O10 | 96.90(9) | | O3 | S2 | O6 | 107.36(14) |
| O5$^{2}$ | Co2 | O10$^{2}$ | 83.10(9) | | O13 | S2 | O3 | 108.59(12) |
| O5 | Co2 | O10 | 83.10(9) | | O13 | S2 | O6 | 107.79(12) |
| O5 | Co2 | O10$^{2}$ | 96.90(9) | | O14 | S2 | O3 | 111.14(13) |
| O10 | Co2 | O4$^{2}$ | 96.21(8) | | O14 | S2 | O6 | 110.23(14) |
| O10$^{2}$ | Co2 | O4 | 96.20(8) | | O14 | S2 | O13 | 111.58(13) |
| O10 | Co2 | O4 | 83.79(8) | | Co3 | O1 | Co1 | 98.03(9) |
| O10$^{2}$ | Co2 | O4$^{2}$ | 83.80(8) | | Co5$^{1}$ | O1 | Co1 | 98.87(10) |
| O10 | Co2 | O10$^{2}$ | 180.0 | | Co5$^{1}$ | O1 | Co3 | 100.95(9) |
| O1$^{3}$ | Co3 | O1 | 180.0 | | Co1$^{1}$ | O2 | Co1 | 99.27(9) |
| O1 | Co3 | O6 | 85.68(8) | | Co1$^{1}$ | O2 | Co5$^{1}$ | 104.39(10) |
| O1$^{3}$ | Co3 | O6$^{3}$ | 85.68(8) | | Co5$^{1}$ | O2 | Co1 | 97.91(9) |
| O1$^{3}$ | Co3 | O6 | 94.32(8) | | Co2 | O4 | Co1 | 90.25(7) |
| O1 | Co3 | O6$^{3}$ | 94.32(8) | | Co5 | O4 | Co1 | 91.12(8) |
| O1$^{3}$ | Co3 | O11$^{3}$ | 82.93(9) | | Co5 | O4 | Co2 | 88.37(7) |
| O1$^{3}$ | Co3 | O11 | 97.07(9) | | S1 | O4 | Co1 | 125.31(11) |
| O1 | Co3 | O11 | 82.93(9) | | S1 | O4 | Co2 | 124.60(14) |
| O1 | Co3 | O11$^{3}$ | 97.07(9) | | S1 | O4 | Co5 | 126.02(14) |
| O6 | Co3 | O6$^{3}$ | 180.0 | | Co2 | O5 | Co4 | 98.04(9) |
| O11$^{3}$ | Co3 | O6$^{3}$ | 97.18(8) | | Co2 | O5 | Co5$^{2}$ | 99.83(8) |
| O11 | Co3 | O6 | 97.18(8) | | Co5$^{2}$ | O5 | Co4 | 97.83(9) |
| O11 | Co3 | O6$^{3}$ | 82.82(8) | | Co3 | O6 | Co4$^{3}$ | 90.62(8) |
| O11$^{3}$ | Co3 | O6 | 82.82(8) | | Co3 | O6 | Co5$^{1}$ | 87.82(7) |
| O11$^{3}$ | Co3 | O11 | 180.0 | | Co5$^{1}$ | O6 | Co4$^{3}$ | 89.82(8) |
| O5 | Co4 | O6$^{3}$ | 170.57(7) | | S2 | O6 | Co3 | 127.07(15) |
| O10 | Co4 | O5 | 80.11(9) | | S2 | O6 | Co4$^{3}$ | 123.55(12) |
| O10 | Co4 | O6$^{3}$ | 94.39(9) | | S2 | O6 | Co5$^{1}$ | 126.41(15) |
| O11 | Co4 | O5 | 105.86(10) | | Co1 | O10 | Co4 | 97.25(9) |
| O11 | Co4 | O6$^{3}$ | 80.75(9) | | Co2 | O10 | Co1 | 103.89(10) |
| O11 | Co4 | O10 | 82.14(8) | | Co2 | O10 | Co4 | 98.66(10) |
| O11 | Co4 | O12 | 97.42(8) | | Co3 | O11 | Co1 | 98.46(10) |
| O11 | Co4 | O12$^{4}$ | 173.48(9) | | Co4 | O11 | Co1 | 98.83(10) |
| O12$^{4}$ | Co4 | O5 | 80.36(9) | | Co4 | O11 | Co3 | 105.33(10) |
| O12 | Co4 | O5 | 103.95(9) | | Co4 | O12 | Co4$^{4}$ | 97.27(9) |
| O12 | Co4 | O6$^{3}$ | 81.47(9) | | Co5$^{6}$ | O12 | Co4 | 104.45(10) |
| O12$^{4}$ | Co4 | O6$^{3}$ | 92.84(8) | | Co5$^{6}$ | O12 | Co4$^{4}$ | 98.87(10) |
| O12$^{4}$ | Co4 | O10 | 97.23(8) | | N1 | C1 | C1$^{7}$ | 110.6(3) |
| O12 | Co4 | O10 | 175.85(8) | | N2 | C2 | C2$^{8}$ | 110.5(3) |
| O12 | Co4 | O12$^{4}$ | 82.73(9) | | | | | |

Symmetry related positions: $^{1}$1-*x*,2-*y*,-*z*; $^{2}$1-*x*,1-*y*,-*z*; $^{3}$-*x*,2-*y*,-*z*; $^{4}$-*x*,1-*y*,-*z*; $^{5}$1+*x*,+*y*,+*z*; $^{6}$-1+*x*,+*y*,+*z*; $^{7}$1-*x*,-*y*,1-*z*; $^{8}$-*x*,1-*y*,1-*z*

## Section 3: Atomic force microscopy

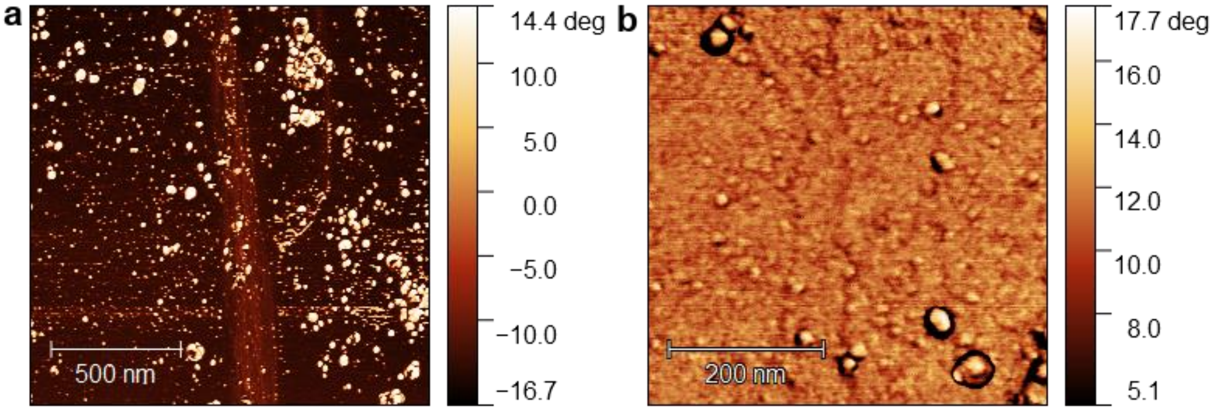


**Fig. S2 | Atomic force microscopy phase images of $Co_4(OH)_6(SO_4)_2[enH_2]$.** Phase images corresponding **a** to the topography image shown in Fig. 1d and **b** to that of Fig. 1e of the main text, respectively.

## Section 4: Powder x-ray diffraction under applied magnetic field

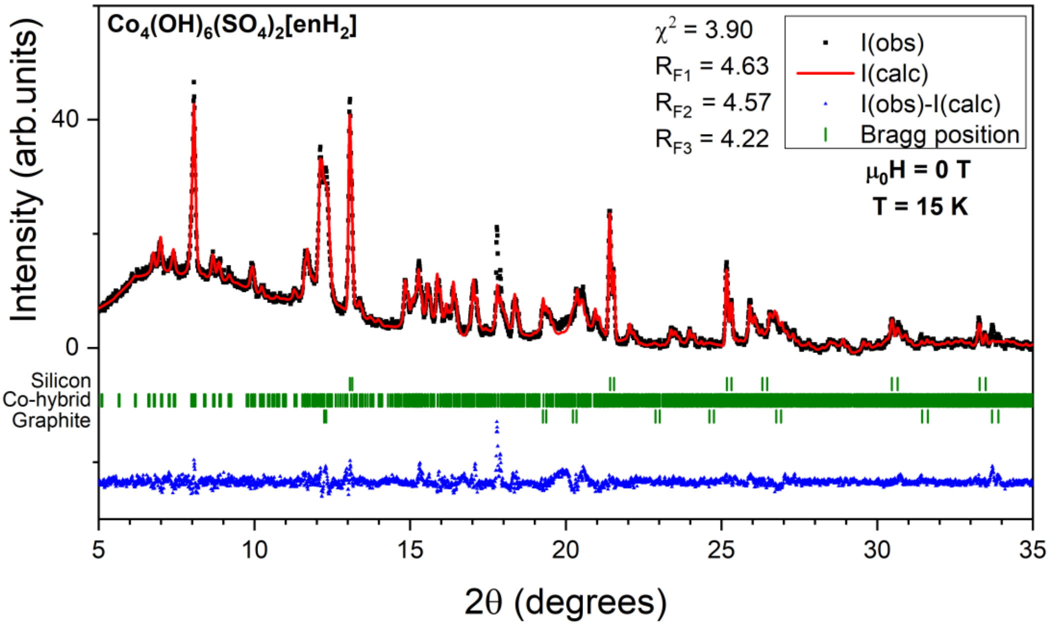


**Fig. S3 | Rietveld refinement of powder x-ray diffraction data of $Co_4(OH)_6(SO_4)_2[enH_2]$ collected at 15 K in zero applied magnetic field.** The fit is based on the structural model determined at 107 K by single-crystal XRD. The black squares indicate the experimentally observed intensity ($I(obs)$), the red line indicates the calculated intensity ($I(calc)$), the blue triangles indicate the difference between the observed and calculated intensities ($I(obs) - I(calc)$) and the green markers indicate the expected peak positions for silicon, $Co_4(OH)_6(SO_4)_2[enH_2]$ and graphite. $R_{F1,}$ $R_{F2}$ and $R_{F3}$ denote the determined $R_F$ factors for the silicon, $Co_4(OH)_6(SO_4)_2[enH_2]$ and graphite phases respectively.

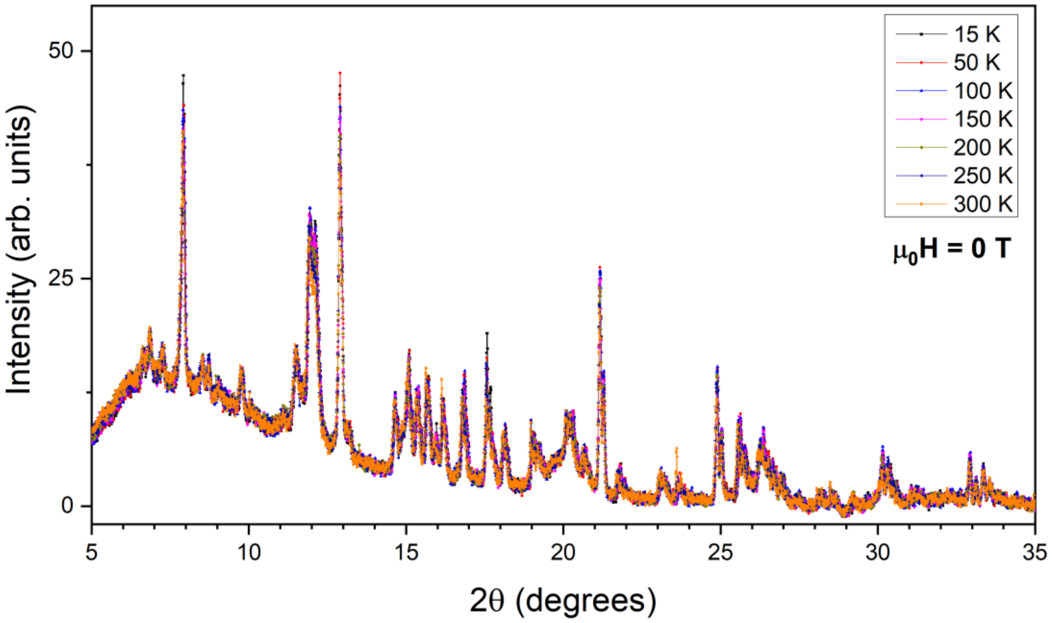


**Fig. S4 | Powder x-ray diffraction data for $Co_4(OH)_6(SO_4)_2[enH_2]$ obtained in zero applied magnetic field, measured between 300 K and 15 K.** The peak shifts that are observed with changing temperature are due to thermal expansion effects.

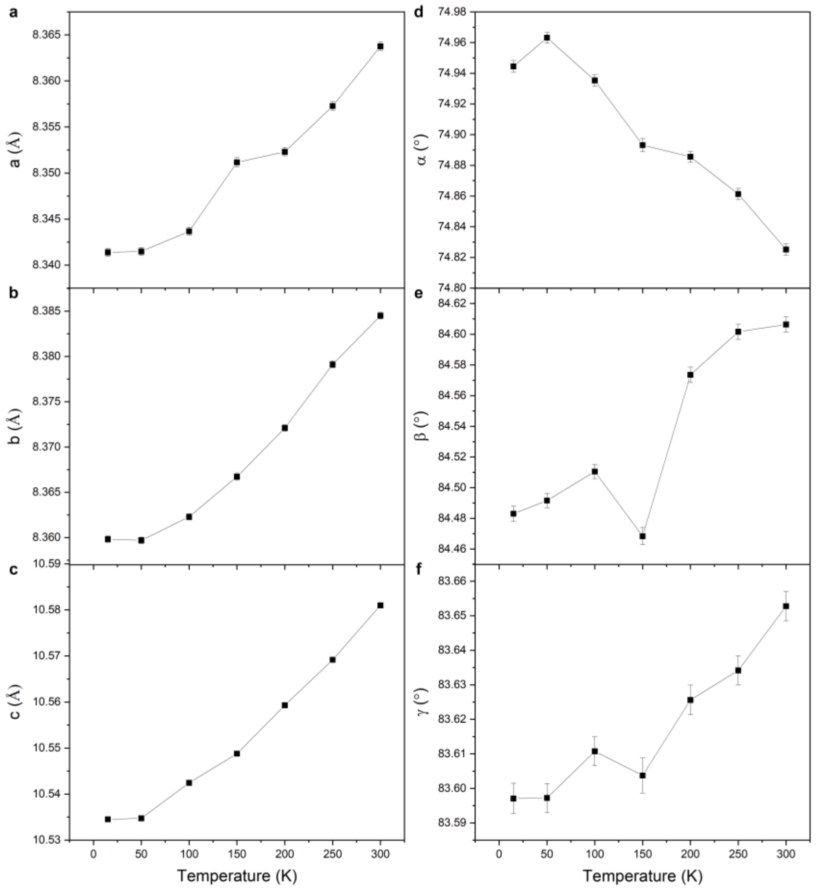


**Fig. S5 | Refined unit cell parameters as a function of temperature for $Co_4(OH)_6(SO_4)_2[enH_2]$ in zero applied magnetic field.** Data were measured on cooling from 300 K to 15 K. Error bars determined by the fitting software are shown.

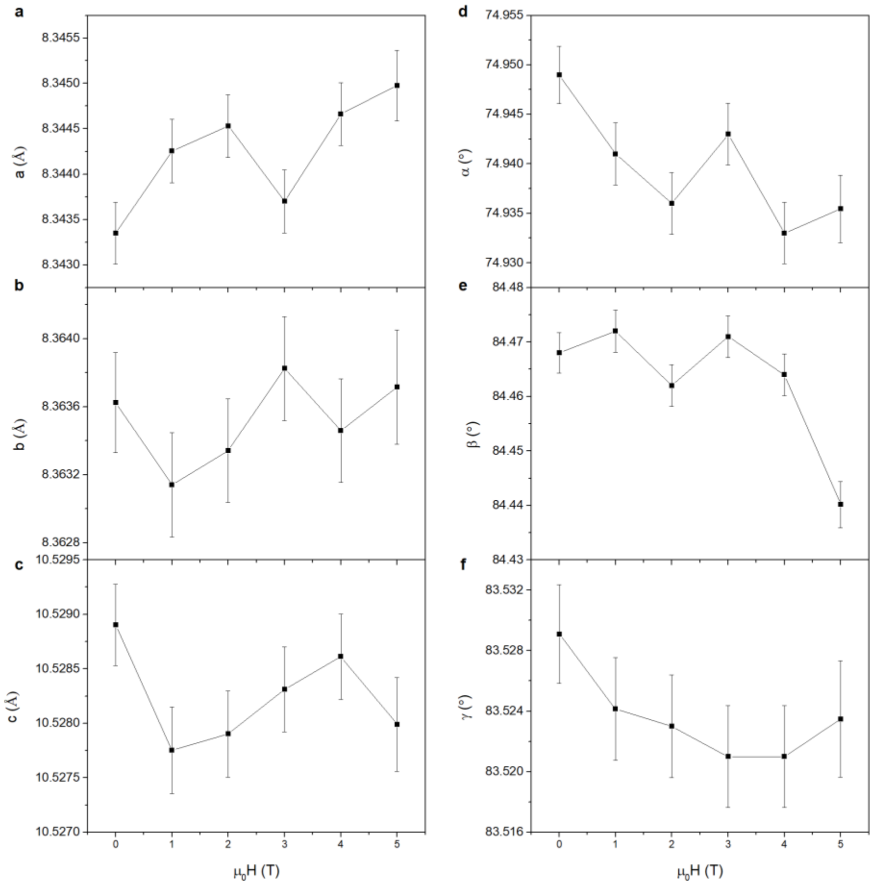

**Fig. S6 | Refined unit cell parameters of $Co_4(OH)_6(SO_4)_2[enH_2]$ as a function of applied magnetic field at 15 K.** Error bars determined by the fitting software are shown.

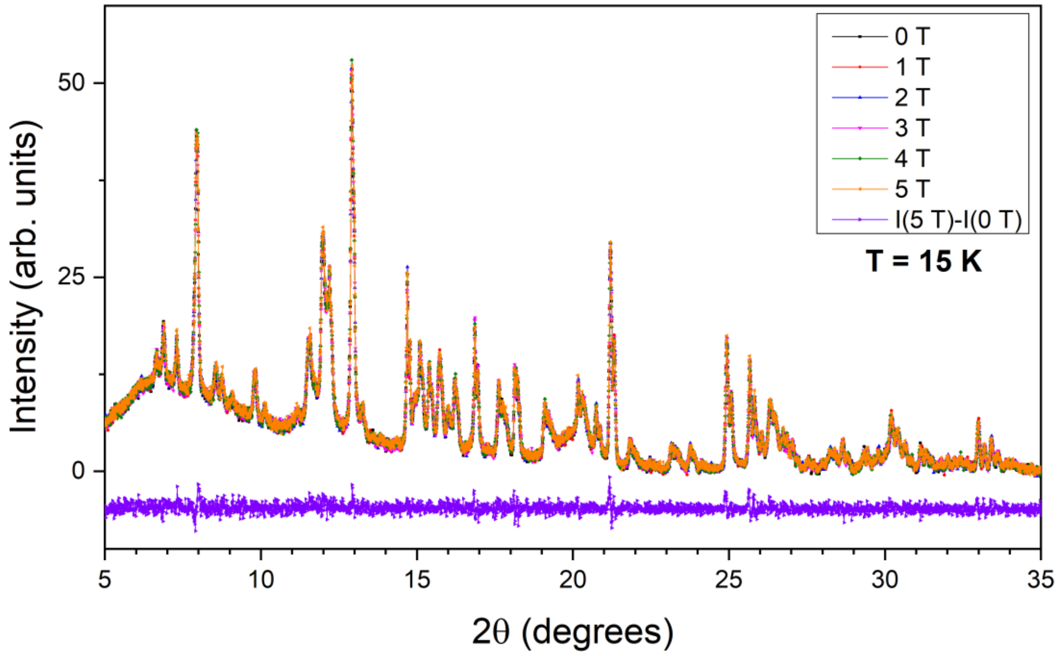


**Fig. S7 | Powder x-ray diffraction data for $Co_4(OH)_6(SO_4)_2[enH_2]$ collected at 15 K in applied magnetic fields from 0 to 5 T.** The difference between the diffraction profiles measured in applied magnetic fields of 5 T and 0 T is plotted below the experimental data.

# Section 5: Magnetometry

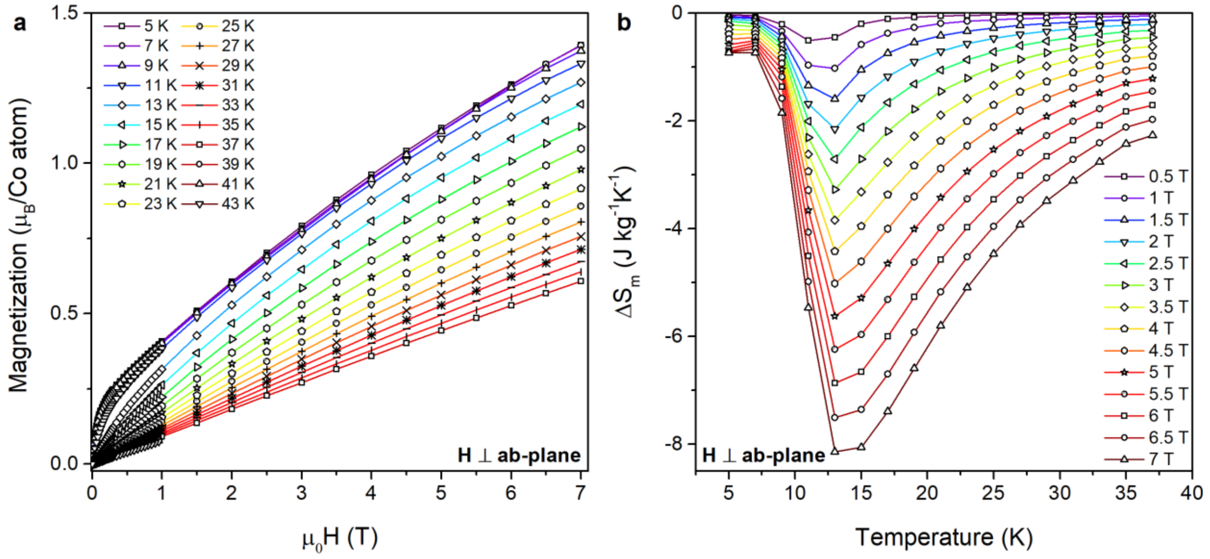


**Fig. S8 | Magnetization versus applied field and entropy change of $Co_4(OH)_6(SO_4)_2[enH_2]$. a** Magnetization measured perpendicular to the *ab*-plane, plotted versus applied magnetic field in the temperature range 5 - 43 K. **b** Corresponding magnetic entropy change $\Delta S_M$ versus temperature.

# Section 6: Critical scaling analysis

The isothermal magnetization measurements and the calculated magnetic entropy change described in Section 2B of the main text can be used to study the critical behavior of $Co_4(OH)_6(SO_4)_2[enH_2]$ near its magnetic ordering temperature. The Banerjee criterion can be applied to the Arrott plot ($H/M$ plotted against $M^2$) shown in Fig. S9a, where the criterion for a second-order magnetic phase transition is fulfilled when all slopes have positive gradients [1]. As an alternative to the common use of the Banerjee criterion, Law et al. proposed that the order of the magnetic phase transition can be determined from the temperature and field dependent critical exponent $n$ [2], which is related to the scaling behavior of the magnetic entropy change with the magnetic field via the proportionality:

$$\Delta S_M^{\mathrm{Pk}} \propto H^n$$

In short, the quantitative criterion proposed by Law et al. states that a for a first-order phase transition, $n$ will exhibit values larger than 2 in the critical region. For a second-order phase transition, $n$ will only exhibit values equal to or smaller than 2 over the entire temperature range. For temperatures much higher than the ordering temperature, $n$ will tend to the paramagnetic value of 2 and for temperatures well below the ordering temperature, $n$ will tend to 1. Figure S9b shows the experimental temperature and field dependent behavior of $n$ in the critical temperature region. Most importantly, for all applied fields $n$ does not reach values larger than 2 over the entire experimental temperature range. For $\mu_0 H\ = 0.5$ T and with decreasing temperature the exponent $n$ decreases from 2 to a minimum around 13 K after which a small peak is observed. The fulfillment of both criteria provides strong evidence for the second-order nature of the phase transition and is consistent with the lack of thermal hysteresis (Fig. 2a, main text) and structural changes observed in our temperature and field dependent magnetization and PXRD data (Figs. S3-S7). Thus, the critical scaling analysis described below is valid.

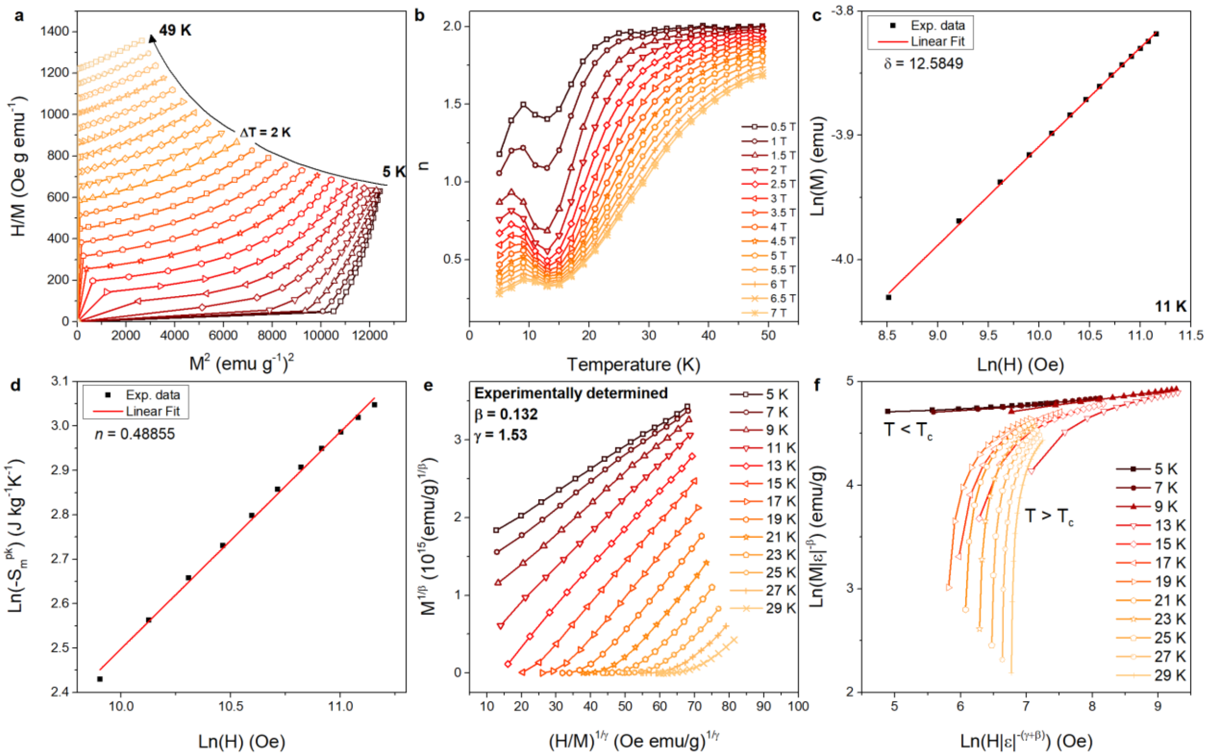


**Fig. S9 | Critical scaling analysis for $Co_4(OH)_6(SO_4)_2[enH_2]$. a** Arrott plot and **b** critical exponent $n$ plotted against temperature. **c, d** Logarithm of the magnetization and peak magnetic entropy change respectively, plotted against the logarithm of the applied magnetic field. The red lines indicate linear fits to the data. **e** Modified Arrott-Noakes plot constructed using the determined critical exponents. **f** Universal curve constructed using the determined critical exponents, showing the formation of two distinct branches for $T > T_c$ (open symbols) and $T < T_c$ (filled symbols).

The critical behavior associated with a second-order phase transition is characterized by a set of related critical exponents according to the scaling hypothesis [3]. The critical exponents $\beta$, $\gamma$ and $\delta$ are associated with the behavior of the spontaneous magnetization ($M_S$) (the order parameter), the inverse initial susceptibility ($\chi_0^{-1}$) and the magnetization ($M(H)$) at $T_c$ and are defined by the following relations [4,5]:

$$M_S(T) = M_0|\varepsilon|^{\beta}, \qquad \varepsilon < 0, \qquad T < T_c,$$
$$\chi_0^{-1}(T) = \left(\frac{h_0}{M_0}\right)|\varepsilon|^{\gamma}, \qquad \varepsilon > 0, \qquad T > T_c,$$
$$M = DH^{\frac{1}{\delta}}, \qquad \varepsilon = 0, \qquad T = T_c$$

Here, $h_0$ and $D$ are the critical amplitudes and $\varepsilon$ is the reduced temperature $(T - T_c)/T_c$. The exponent $n$ is related to the other critical exponents via the following relation:

$$n = 1 + \frac{1}{\delta}\left(1 - \frac{1}{\beta}\right)$$

By fitting a linear curve to the $\ln(M)$ versus $\ln(H)$ data at $T_c$ (11 K data in this case) we are able to obtain $1/\delta$ from the slope (Fig. S9c). From a linear fit to the $\ln(S_M^{Pk})$ versus $\ln(H)$ data we obtain the exponent $n$ (Fig. S9d). Then through the equation above we are able to obtain the $\beta$ exponent. $\gamma$ can be obtained through the Widom scaling relationship:

$$\delta = 1 + \gamma/\beta$$

The experimentally determined critical exponents are found to be $n = 0.489$, $\delta = 12.585$, $\beta = 0.134$ and $\gamma = 1.53$. A modified Arrot-Noakes plot was constructed from the extracted exponents (Fig. S9e), which shows a set of nearly parallel curves that are linear close to the ordering temperature. The 11 K curve, which lies closest to the ordering temperature, intercepts the origin. The construction of a universal curve (Fig. S9f) shows the formation of two separate branches above and below $T_c$, validating the above approach.

The critical exponents lie closest to the expected values for the 2D XY model with exponents $\beta = 1/8$, $\gamma = 7/4$ and $\delta = 15$ [6]. Our results are compared to modified Arrott-Noakes plots constructed from the critical exponents of various models (2D XY, 3D Ising, 3D Heisenberg, 3D XY and mean field theory) in Fig. S10a-f. It is also apparent here that the 2D XY model yields the most suitable description of the critical behavior in this compound. More accurate critical exponents could be determined through higher resolution $M(\mu_0 H)$ measurements close to $T_c$ as well as other experimental techniques such as muon spin spectroscopy.

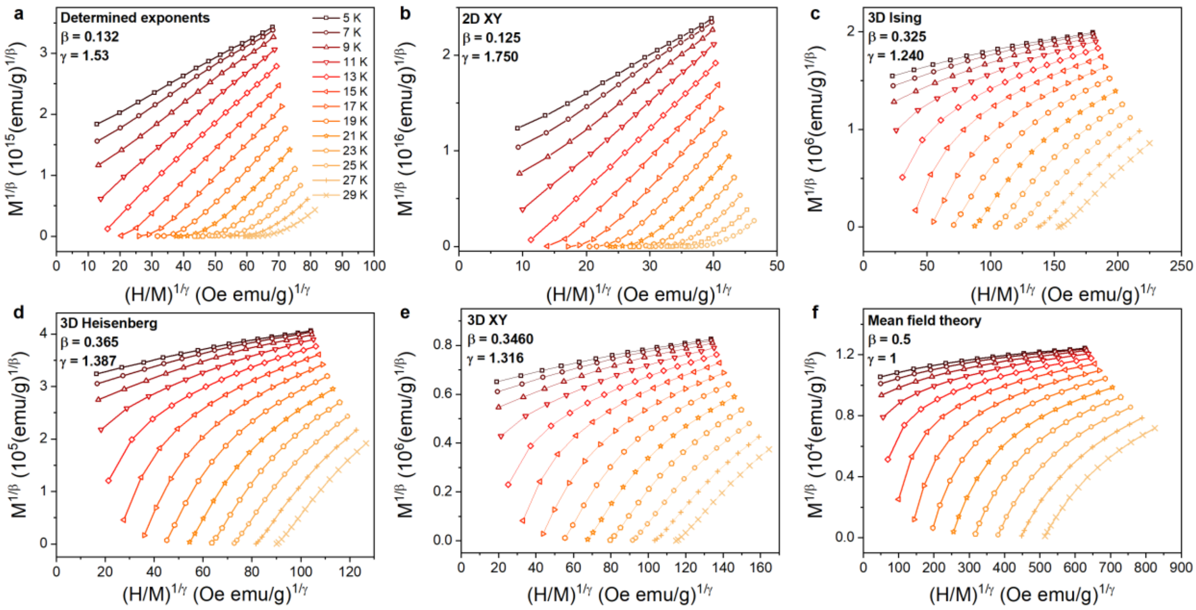


**Fig. S10 | Modified Arrott-Noakes plots for different magnetic models.** The plots are constructed using the critical exponents from **a** the experimental data, **b** the 2D XY model, **c** the 3D Ising model, **d** the 3D Heisenberg model, **e** the 3D XY model and **f** the mean field theory model.

## Section 7: Comparison of $\Delta S_M$ from $C_p$ and isothermal $M(\mu_0 H)$ measurements

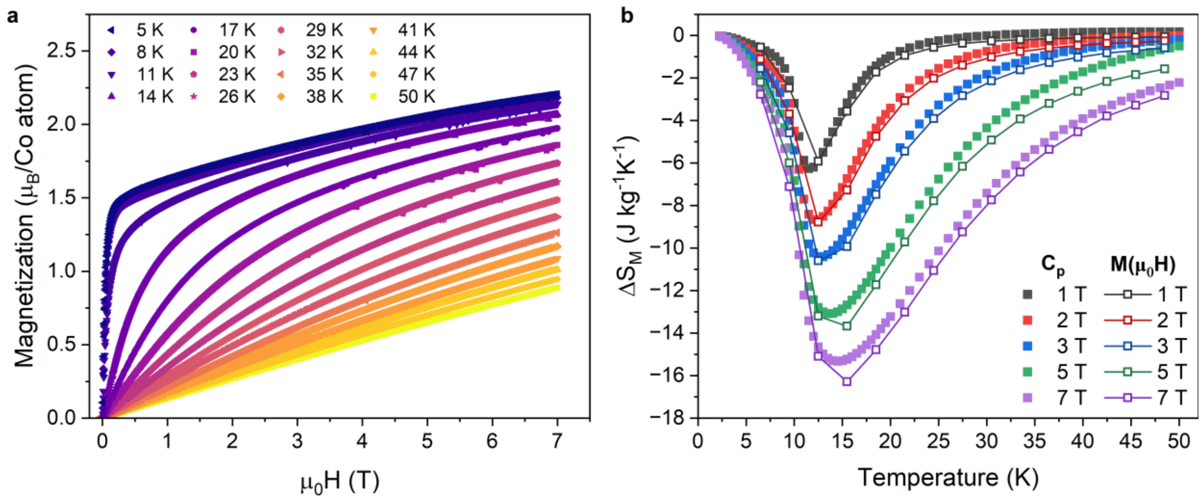


**Fig. S11 | Magnetization versus applied field and comparison of entropy change extracted from magnetization and heat capacity measurements. a** Magnetic field dependence of magnetization measured isothermally from 5 - 50 K on the same polycrystalline sample that was used for the heat capacity measurements shown in Fig. 3a. **b** Comparison of the $\Delta S_M$ values extracted from the $M(\mu_0 H)$ curves shown in (**a**) and from the heat capacity curves shown in Figs. 3a and b.

## Section 8: Direct measurement of $\Delta T_{\mathrm{ad}}$ in pulsed magnetic fields

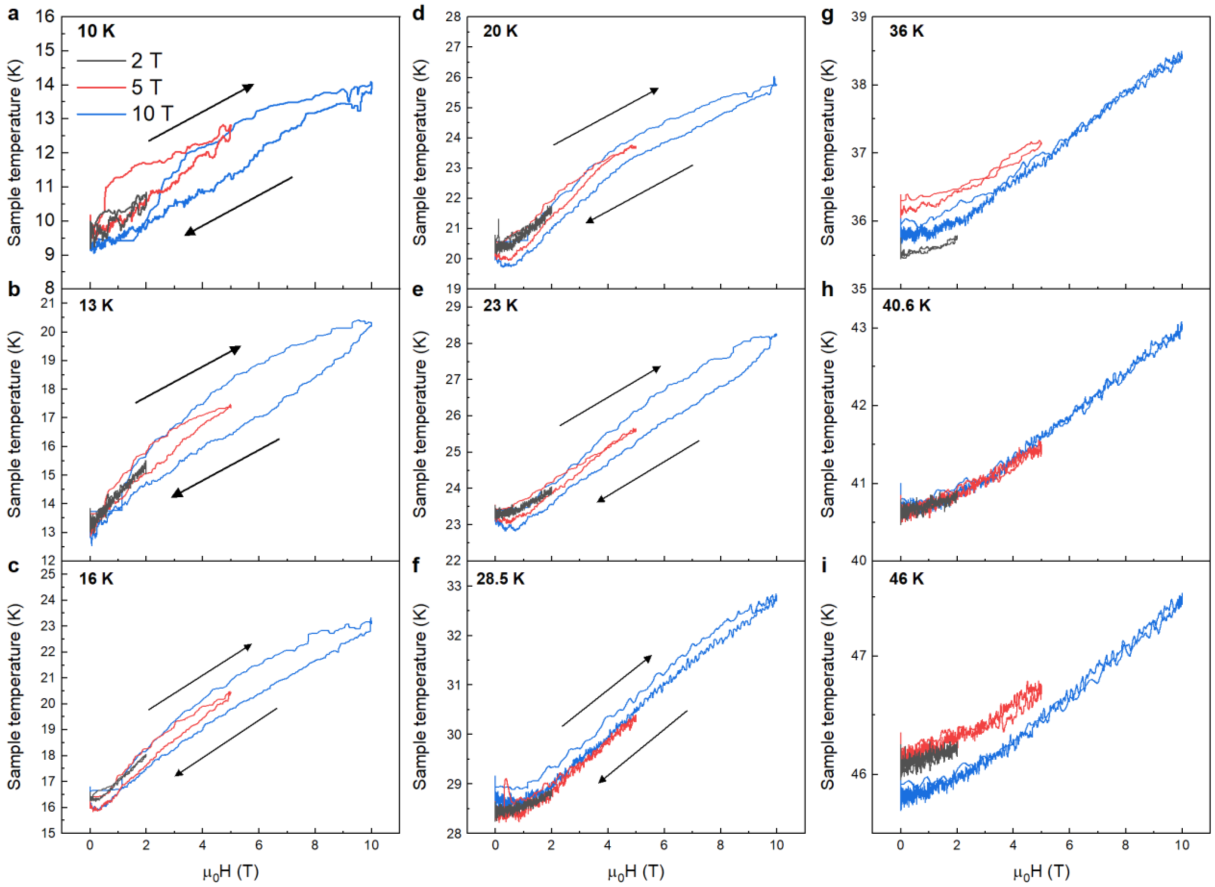


**Fig. S12 | Measured sample temperature plotted against applied magnetic field in pulsed magnetic fields up to 10 T.** The arrows indicate the sample temperature measured upon the application or removal of the magnetic field. For the sake of clarity, the data presented here have been smoothed using a median filter due to the presence of large localized noise spikes in the raw data.

## Section 9: Comparison of magnetocaloric properties

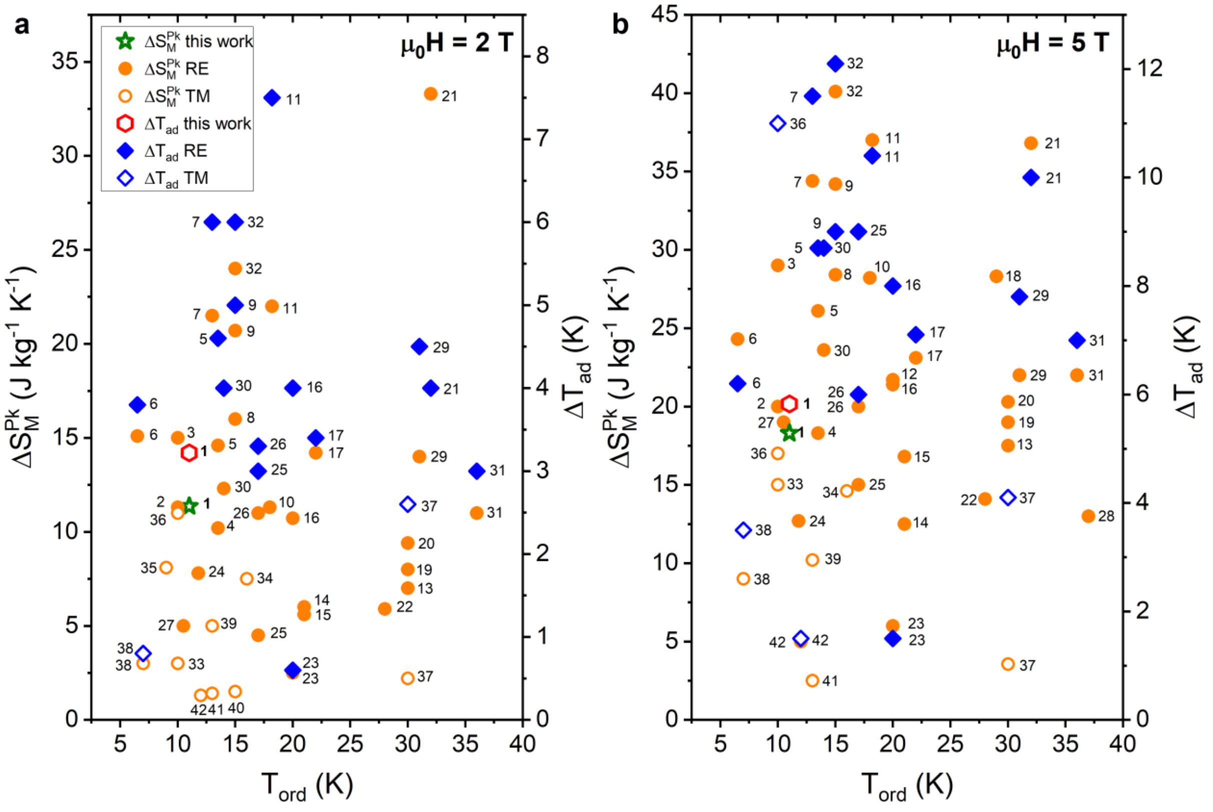


**Fig. S13 | Reported values of magnetic entropy and adiabatic temperature change for various prospective magnetocaloric materials and $Co_4(OH)_6(SO_4)_2[enH_2]$.** The values are plotted against their respective magnetic ordering temperatures ($T_{ord}$), for **a** $\Delta\mu_o H = 2$ T and **b** $\Delta\mu_o H = 5$ T. The filled symbols indicate that the compound contains a rare-earth element and the open symbols indicate that the compound is based solely on transition-metal elements. The numeric labels refer to the compounds listed in Table S6.

**Table S6 | List of prospective magnetocaloric compounds and their labels used in Figs. 4 and S13a, b.**

| Compound name | Label | | Compound name | Label |
|---|---|---|---|---|
| $Co_4(OH)_6(SO_4)_2[enH_2]$[this work] | 1 | | HoNi[15] | 22 |
| HoPd[8] | 2 | | PrNi[16] | 23 |
| ErNi[10] | 3 | | $HoCo_3B_2$[18] | 24 |
| $Er_{12}Co_7$[12] | 4 | | $GdPd_2Si$[20] | 25 |
| $HoNi_2$[14] | 5 | | $Er_3Ni_2$[22] | 26 |
| $ErNi_2$[14] | 6 | | $HoCu_2$[24] | 27 |
| $ErAl_2$[17] | 7 | | DyCoAl[26] | 28 |
| $GdCoC_2$[19] | 8 | | DyNiAl[28] | 29 |
| TmGa[21] | 9 | | HoNiAl[30] | 30 |
| HoN[23] | 10 | | $Ho_3Ni_2$[22] | 31 |
| EuS[25] | 11 | | $HoB_2$[32] | 32 |
| HoNiIn[27] | 12 | | $Co(OH)_2$[33] | 33 |
| $Ho_2Cu_2In$[29] | 13 | | $CrCl_3$[35] | 34 |
| $Ho_2Au_2In$[29] | 14 | | $Co_2(OH)_{4-x}Cl_x$[36] | 35 |
| DyN[31] | 15 | | $Co_3V_2O_8$[37] | 36 |
| $DyNi_2$[17] | 16 | | MnSi[38] | 37 |
| ErFeSi[34] | 17 | | $Ni(C_4H_3O_4)_2(H_2O)_4$[39] | 38 |
| $HoAl_2$[7] | 18 | | $\{(H_3O)_2[Mn_3(SO_4)_3(\mu_3\text{-}OH)_2(H_2O)_2]\}_n$[40] | 39 |
| $Ho_{12}Co_7$[9] | 19 | | $(C_6H_5CH_2CH_2NH_3)_2CuCl_4$[41] | 40 |
| $Ho_2Cu_2Cd$[11] | 20 | | $(PMA)_2CuCl_4$[42] | 41 |
| $ErCo_2$[13] | 21 | | $(CH_3NH_3)_2CuCl_4$[43] | 42 |

# References


1. Banerjee, B. K. On a generalised approach to first and second order magnetic transitions. *Phys. Lett.* **12**, 16–17 (1964).
2. Law, J. Y. *et al.* A quantitative criterion for determining the order of magnetic phase transitions using the magnetocaloric effect. *Nature Commun.* **9**, 2680 (2018).
3. Stanley, H. E. Scaling, universality, and renormalization: Three pillars of modern critical phenomena. *Rev. Modern Phys.* **71**, S358–S366 (1999).
4. Fan, J. *et al.* Critical properties of the perovskite manganite $La_{0.1}Nd_{0.6}Sr_{0.3}MnO_3$. *Phys. Rev. B* **81**, 144426 (2010).
5. Pełka, R. *et al.* Magnetic systems at criticality: Different signatures of scaling. *Acta Phys. Polon. A* **124**, 977–988 (2013).
6. Kosterlitz, J. M. The critical properties of the two-dimensional xy model. *J. Phys. C : Solid State Phys.* **7**, 1046–1060 (1974).
7. Hashimoto, T. *et al.* New application of complex magnetic materials to the magnetic refrigerant in an Ericsson magnetic refrigerator. *J. Appl. Phys.* **62**, 3873–3878 (1987).
8. Mo, Z. J. *et al.* Magnetic properties and magnetocaloric effects in HoPd intermetallic. *Chinese Phys. B* **24**, 037503 (2015).
9. Zheng, X. Q. *et al.* Giant magnetocaloric effect in $Ho_{12}Co_7$ compound. *Appl. Phys. Lett.* **102**, 022421 (2013).
10. Kumar, P., Suresh, K. G., Nigam, A. K. & Gutfleisch, O. Large reversible magnetocaloric effect in RNi compounds. *J. Phys. D: Appl. Phys.* **41**, 245006 (2008).
11. Yi, Y., Li, L., Su, K., Qi, Y. & Huo, D. Large magnetocaloric effect in a wide temperature range induced by two successive magnetic phase transitions in $Ho_2Cu_2Cd$ compound. *Intermetallics* **80**, 22–25 (2017).
12. Zheng, X. *et al.* Large magnetocaloric effect in $Er_{12}Co_7$ compound and the enhancement of $\delta T_{FWHM}$ by Ho-substitution. *J. Alloys Compd.* **680**, 617–622 (2016).
13. Wada, H., Tanabe, Y., Shiga, M., Sugawara, H. & Sato, H. Magnetocaloric effects of Laves phase $Er(Co_{1-x}Ni_x)_2$ compounds. *J. Alloys Compd.* **316**, 245–249 (2001).
14. Ćwik, J., Koshkid'ko, Y., Nenkov, K., Tereshina, E. A. & Rogacki, K. Structural, magnetic and magnetocaloric properties of $HoNi_2$ and $ErNi_2$ compounds ordered at low temperatures *J. Alloys Compd.* **735**, 1088–1095 (2018).
15. Rajivgandhi, R., Arout Chelvane, J., Quezado, S., Malik, S. K. & Nirmala, R. Effect of rapid quenching on the magnetism and magnetocaloric effect of equiatomic rare earth intermetallic compounds RNi (R = Gd, Tb and Ho). *J. Magn. Magn. Mater.* **433**, 169–177 (2017).
16. Pecharsky, O. *et al.* Preparation, crystal structure, heat capacity, magnetism, and the magnetocaloric effect of $Pr_5Ni_{1.9}Si_3$ and PrNi. *Phys. Rev. B* **68**, 134452 (2003).
17. von Ranke, P. J., Pecharsky, V. K. & Gschneidner, K. A. Influence of the crystalline electrical field on the magnetocaloric effect of $DyAl_2$, $ErAl_2$, and $DyNi_2$. *Phys. Rev. B* **58**, 12110 (1998).
18. Zheng, X. Q. *et al.* Magnetic properties and magnetocaloric effect of $HoCo_3B_2$ compound. *AIP Adv.* **8**, 056432 (2018).
19. Meng, L. *et al.* Magnetic properties and giant reversible magnetocaloric effect in $GdCoC_2$. *RSC Adv.* **6**, 74765–74768 (2016).
20. Rawat, R. & Das, I. Magnetocaloric and magnetoresistance studies of $GdPd_2Si$. *J. Phys.: Condens. Mat.* **13**, L57–L63 (2001).
21. Mo, Z. J. *et al.* Low field induced giant magnetocaloric effect in TmGa compound. *Appl. Phys. Lett.* **103**, 052409 (2013).
22. Dong, Q. Y., Chen, J., Shen, J., Sun, J. R. & Shen, B. G. Magnetic properties and magnetocaloric effects in $R_3Ni_2$ (R = Ho and Er) compounds. *Appl. Phys. Lett.* **99**, 132504 (2011).
23. Yamamoto, T. A., Nakagawa, T., Sako, K., Arakawa, T. & Nitani, H. Magnetocaloric effect of rare earth mono-nitrides, TbN and HoN. *J. Alloys Compd.* **376**, 17–22 (2004).

24. Karmakar, S. K., Giri, S. & Majumdar, S. Observation of large low temperature magnetocaloric effect in $HoCu_2$. *J. Appl. Phys.* **117**, 193904 (2015).
25. Li, D. X. *et al.* Large reversible magnetocaloric effect in ferromagnetic semiconductor EuS. *Solid State Commun.* **193**, 6–10 (2014).
26. Chelvane, J. A. *et al.* Magnetic structure and magnetic entropy change in the intermetallic compound DyCoAl. *J. Appl. Phys.* **107**, 09A906 (2010).
27. Zhang, H. *et al.* Magnetic properties and magnetocaloric effects in $Gd_{1-x}Ho_xNiIn$ intermetallic compounds. *Solid State Commun.* **152**, 1734–1738 (2012).
28. Kaštil, J., Javorský, P. & Andreev, A. V. Anisotropy of the magnetocaloric effect in DyNiAl. *J. Magn. Magn. Mater.* **321**, 2318–2321 (2009).
29. Li, L. *et al.* Magnetic properties and large magnetocaloric effect in $Ho_2Cu_2In$ and $Ho_2Au_2In$ compounds. *J. Mater. Sci.* **51**, 5421–5426 (2016).
30. Singh, N. K., Suresh, K. G., Nirmala, R., Nigam, A. K. & Malik, S. K. Effect of magnetic polarons on the magnetic, magnetocaloric, and magnetoresistance properties of the intermetallic compound HoNiAl. *J. Appl. Phys.* **101**, 093904 (2007).
31. Nakagawa, T., Sako, K., Arakawa, T. & Yamamoto, T. A. Magnetocaloric effect of mononitride containing gadolinium and dysprosium $Gd_xDy_{1-x}N$. *J. Alloys Compd.* **364**, 53–58 (2004).
32. de Castro, P. B. *et al.* Machine-learning-guided discovery of the gigantic magnetocaloric effect in $HoB_2$ near the hydrogen liquefaction temperature. *NPG Asia Mater.* **12**, 35 (2020).
33. Liu, X. H. *et al.* Giant reversible magnetocaloric effect in cobalt hydroxide nanoparticles. *Appl. Phys. Lett.* **93**, 202502 (2008).
34. Zhang, H. *et al.* Large reversible magnetocaloric effects in ErFeSi compound under low magnetic field change around liquid hydrogen temperature. *Appl. Phys. Lett.* **102**, 092401 (2013).
35. Mondal, S., Midya, A., Patidar, M. M., Ganesan, V. & Mandal, P. Magnetic and magnetocaloric properties of layered van der Waals $CrCl_3$. *Appl. Phys. Lett.* **117**, 092405 (2020).
36. Song, S. -H., Alonso, J. A., Fernández-Díaz, M. T. & Lim, S. H. Crystal structure and magnetic and magnetocaloric properties of cobalt hydroxychlorides $Co_2(OH)_{4-x}Cl_x$. *Eur. J. Inorg. Chem.* **2017***,* 2289-2294 (2017).
37. Mukherjee, S. *et al.* Linear magnetoelectric coupling without long-range magnetic order and rare-earth-free large magnetocaloric effect in $Co_3V_2O_8$. *Phys. Rev. B* **109**, 014418 (2024).
38. Arora, P., Chattopadhyay, M.K. & Roy, S.B. Magnetocaloric effect in MnSi. *Appl. Phys. Lett.* **91**, 062508 (2007).
39. Danylchenko, P. *et al.* Experimental study of magnetocaloric effect in tetraaquabis(hydrogen maleato)nickel(II), $[Ni(C_4H_3O_4)_2(H_2O)_4]$—a potential realization of a spin-1 spatially anisotropic square lattice with ferromagnetic interactions. *Magnetochemistry* **8**, 106 (2022).
40. Wei, W., Xie, R-K., Du, S-W., Tian, C-B., Chai, G-L. Synthesis, structure, magnetocaloric effect and DFT calculations of a $Mn^{II}$ cluster-based inorganic coordination polymer. *J. Alloys Compd.* **878**, 160353 (2021).
41. Park, G. *et al.* Critical behavior of quasi-2D organic-inorganic halide perovskite $(C_6H_5CH_2CH_2NH_3)_2CuCl_4$ single crystals. *Current Appl. Phys.* **35**, 24-31 (2022).
42. Septiany, L. & Blake, G.R. Magnetocaloric effect and critical behavior in arylamine-based copper chloride layered organic-inorganic perovskite *J. Magn. Magn. Mater.* **542**, 168598 (2022).
43. Ma, Y. *et al.* Magnetocaloric effect in the layered organic–inorganic hybrid $(CH_3NH_3)_2CuCl_4$ *Chinese Phys. B* **27**, 027501 (2018).
